# Empowering human research ethics committees to review genomics applications: evaluating the utility of a custom online education resource

**DOI:** 10.1038/s41431-025-01846-5

**Published:** 2025-04-17

**Authors:** Ella McGahan, Jennifer Berkman, David Milne, Bronwyn Terrill, Russell Gear, Susan Gardiner, Lisa Eckstein, Dianne Nicol, Natalie Taylor, Ingrid Winship, Rebekah McWhirter, Amy Nisselle, Jason Lodge, Aideen McInerney-Leo

**Affiliations:** 1 Frazer Institute, University of Queensland, Dermatology Research Centre, Brisbane, QLD Australia; 2 Metro South Human Research Ethics Committee, Brisbane, QLD Australia; 3 Garvan Institute of Medical Research, University of New South Wales, Sydney, NSW Australia; 4 Murdoch Children’s Research Institute, Melbourne, VIC Australia; 5 The University of Melbourne, Melbourne, VIC Australia; 6 Royal Melbourne Hospital, Melbourne, VIC Australia; 7 Faculty of Law, University of Tasmania, Hobart, TAS Australia; 8 Bellberry Ltd, Eastwood, SA Australia; 9 Centre for Law and Genetics, University of Tasmania, Hobart, TAS Australia; 10 Faculty of Medicine and Health, University of New South Wales, Sydney, NSW Australia; 11 ANU College of Law, Australian National University, Canberra, ACT Australia; 12 School of Education, University of Queensland, Brisbane, QLD Australia

**Keywords:** Genetics research, Ethics, Translational research

## Abstract

Complex genomic technologies are increasingly utilised in research. However, human research ethics committee (HREC) members lack confidence reviewing genomics applications. This study developed and evaluated the acceptability and utility of an online educational resource on genomics and the ethical considerations for HREC members. Resource development and evaluation was theoretically informed. Qualitative semi-structured interviews with HREC members and subject experts were transcribed and deductively analysed. Participants (*n* = 29) found the content to be comprehensive, appropriately pitched, and optimal in quantity. Most reported the resource was easy to access and intuitive to navigate. HREC members reported improved confidence in reviewing genomics ethics applications and intentions to re-access as needed. Most (*n* = 28/29) would recommend to other HREC members, and some volunteered that they would recommend to researchers. Suggested navigation improvements included a progress bar, active learning elements, and a more clearly visible menu. Content suggestions included more detail on data storage/management and considerations when engaging diverse communities. This is the first study to develop and evaluate a genomic educational resource tailored to ethics committees. Following refinement and quantitative evaluation, it is hoped that this resource will increase HREC member confidence in reviewing genomics ethics applications and the quality of researchers’ submissions.

## Introduction

The scale and complexity of genomic testing, research and the associated data has been exponentially increasing over the past decade [1]. Research genomic testing ranges from genotyping (individual variants and arrays), to next generation sequencing (gene panels, exome sequencing and whole genome sequencing), and/or expression analysis including RNA sequencing [2]. Depending on the research aims, technological and analytical approach, the condition being tested, research setting and scope, target population (e.g., minority groups), whether family members are enroled and whether results are returned, each genomic research project can be associated with diverse ethical considerations (further detailed below). Consequently, it has become increasingly important for ethics review committees such as Human Research Ethics Committees (HRECs) or Institutional Review Boards (IRBs) to be aware of the ethical, legal and social implications (ELSI) of genomics research, and feel empowered to evaluate genomics ethics applications. This understanding is essential to strike the balance between promoting scientific advancements and mitigating participant risk.

Informed consent aims to protect participants’ legal and ethical rights [3], and a well-written participant information and consent form (PICF) clearly communicates study aims, what participation entails and whether participants can opt-in or opt-out, potential outcomes, and ELSI [4]. PICFs for genomic studies should additionally address what types of samples and data (medical, family history etc) will be collected, the nature of genomic technology being used, the analysis being performed, possible result types that may be returned, if any, and the implications of those results [4]. HRECs, or IRBs, review applications relative to the principles of medical ethics (beneficence, non-maleficence, autonomy, and justice) [5], and genomics research presents unique considerations. In terms of beneficence (do good), genomics studies results (if clinically validated) have the potential to explain the aetiology and inheritance of the presenting condition, and/or identify risks for later onset conditions, where appropriate referrals could reduce morbidity and mortality, thereby benefiting both the participant and family. Conversely, the results generated by research have the potential to cause harm, which pertains to non-maleficence (do no harm). Most participants enrol in genomics research with the hope of learning the cause of the primary condition. However, depending on the research aims, technological and analytical approach, genomic research can incur the risk of an ‘incidental’ finding (inadvertently discovered during analysis) [6]. Additionally, some studies will offer to conduct a ‘secondary’ analysis (purposeful data filtering to identify actionable variants unrelated to the primary condition) [7]. Psychologically, depending on personal/family history some results may be more expected and easier to adjust to than others. Furthermore, genetic results are vulnerable to potential genetic discrimination i.e., the differential treatment of a person related to their actual or assumed genetic characteristics or results [8]. Fear of genetic discrimination, especially as it pertains to life insurance underwriting, can inhibit uptake of genomic testing and/or inhibit research participation [9, 10]. The principle of autonomy (self-governance) is complicated in genomics studies by the fact that family members are often invited to participate. This may, overtly or subconsciously, affect an individual’s decision to participate [11]. Furthermore, the shared nature of genetic information means that results may have implications not only for the participant, but also for the nuclear and extended family [12]. Thus, participants may need to share their participation and results with family members, which is a point of distinction with genomic research. Additionally, justice pertains to equality and equity of access to the study, where diverse populations are appropriately represented and benefit from the outcomes [13]. As genomic variants and their associated risks can vary between ancestral populations [14], and biobanks are skewed towards populations of European ancestry [15], there is a risk that poorly-designed genomic research could exacerbate existing health inequities [16]. Moreover, researchers must consider their target population (minority group, regional or international etc.) and conduct research in a manner emphasising consent and consultation, protecting the interests of participants and their data [17].

The National Health and Medical Research Council (NHMRC) certifies HRECs in Australia, of which there are >200 [18]. Committees comprise at least eight members with diverse experience: a chairperson, two laypeople, an allied/medical health professional, a pastoral member, a lawyer, and two researchers [4]. A 2020 survey of Australian HREC members (*n* = 145) reported low-to-moderate confidence around the genomic science in studies and the associated ethical considerations, resulting in significantly higher confidence reviewing non-genomic applications than genomic applications [19]. Furthermore, lay/legal/pastoral HREC members were significantly less confident than scientific/medical members in reviewing genomic applications. Having undertaken any form of genomic education positively predicted confidence and three-quarters of participants agreed that additional genomic education resources would be beneficial, ideally online [19].

We developed a genomics educational resource aiming to empower HRECs to more confidently review genomics ethics applications by increasing knowledge of genomics and the associated ethical considerations. Ultimately, the goal is to promote more efficient and comprehensive assessments and improve the quality of genomic research. To our knowledge, this is the first educational resource focusing on genomic ethical considerations that has been developed specifically for HREC members. This study evaluated the acceptability, perceived utility, and participant satisfaction with the design and format of the educational resource.

## Materials and methods

This project received ethics approval from the University of Queensland (2022/HE002202) and complies with the requirements of the National Statement on Ethical Conduct in Human Research (2023).

### Guiding frameworks

**Fig. 1:**
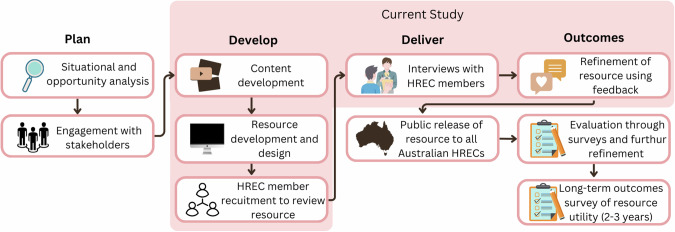
Project specific program logic elements. Specific elements relative to this component of the study have been highlighted in pink.

The development of this resource was guided by the Program Logic Model for Genomics Educational Interventions [20]. In brief, for this study, the programme logic model outlines elements in four steps: plan (situational and opportunity analysis), develop (curriculum and learning design, development of assessment items), deliver (delivery and refinement of resources) and outcomes (see Fig. 1). The accompanying Reporting Item Standards for Education and Evaluation of Genomics (‘RISE2 Genomics’) [20] is a checklist enabling researchers to systematically document the application of the Program Logic Model. The checklist for this study can be found in Supplementary table 1. The development of these educational materials for adults in diverse contexts and learning environments was informed by the Higher Education Learning Framework (HELF) [21]. Principles include ‘learning as becoming’, ‘contextual learning’, ‘deep and meaningful learning’, ‘emotions and learning’, ‘interactive learning’, ‘learning to learn and higher order thinking’, and’ learning challenge and difficulty’.

### Learning objectives

The learning objective was to increase HREC members comfort, confidence, and competence with reviewing genomics applications by (1) developing resources on genomics and genomic technologies which can be understood by lay HREC members and (2) creating resources to convey the diverse ELSI of genomic information.

### Develop

#### Content and resource development and design

The resource (Box 1) was developed in collaboration with an advisory panel consisting of experts in adult and online education, genomics education, bioethics, implementation science, and consumers (i.e., HREC members). Included features and resource delivery were mapped to the relevant HELF principles [21] applicable to a HREC member audience (see Supplementary material 1). All ELSI considerations were mapped relative to and informed by the Australian National Statement on Ethical Conduct in Human Research [4], which includes a chapter (3.3.) specifically addressing ELSI in genomic research. The curriculum was written by AML and the resource was created by EM using the ‘Courses’ feature of the Squarespace website builder. Lecture-style videos were recorded by AML, and authors EM and JB featured in simulation videos. An animated video was also commissioned externally for the resource.

The content was split into five modules that address the knowledge required to understand and review genomics ethics applications (*Genomics 101, Genomics Research Result Types, Ethical Legal and Social Issues, Genomics Technologies and Specific ELSI considerations, and Reviewing a Genomics Application*). Modules included a mixture of recorded lectures, text, figures, animations, videos of simulated discussions, and links to YouTube videos (Supplementary material 2). We estimated a course completion time of 1.5−2 h.

#### Recruitment

A combination of current and previous HREC members, consumer members of the advisory panel, and experts in relevant fields were invited to review the resource between February and June of 2024. HREC members were recruited through contacting HREC chairs, offering to speak at HREC meetings and asking HREC co-ordinators to send emails of invitation to members. All HREC members had free access to the resource, even if they did not wish to participate in the study. In addition, five experts in genomics and/or genomics ethics (‘genomics experts’) were recruited. The invitation and reminder emails (sent 3−4 weeks after the initial email) included a link to the resource, participant information sheet and a link to an online expression of interest form to enter their contact details and consent to participate. Four experts in relevant fields (genomics education and genomics ethics), two of which who also serve on a HREC, were personally invited to participate. All individuals verbally confirmed their consent before commencing an interview.

Box 1 Website address for Resource The HREC Genomics educational resource was published online and is freely accessible through the Integrating Genomics into Medicine Group website: https://www.igmgenomics.com/hrec-course.

### Deliver

#### Interviews

Semi-structured interviews were conducted over Zoom Video Communications, Inc [22]. Interview script (Supplementary material 3) development was guided by the ‘RISE2 Genomics’ reporting standards [23] elements that pertain to the delivery of the educational intervention (which served as the evaluation of the ‘deliver’ stage of the Program Logic Model) and the Kirkpatrick’s framework [24] (evaluating reaction to and impact of the resource). As interviews were conducted immediately after completion of the resource, only the reaction and learning elements of the Kirkpatrick framework were explored. Evaluating the remaining two levels (behaviour and results) is the focus of ongoing research.

Interview questions assessed participant’s prior HREC and genomics experience and confidence, satisfaction with the resource (i.e., ease of navigation and content quality, comprehensiveness, and volume), acceptability as a mode of learning, perceived utility of the resource (changes in confidence and knowledge) and suggestions for improvements. ‘Genomics experience’ was defined as individuals who had undertaken some form of genomics/genomics ethics training (e.g., university degree, short training courses), or had engaged with genomics in a professional setting. Interviews were transcribed and deidentified. All participants were assigned pseudonyms.

### Qualitative data analysis

Content analysis was conducted using a manifest analytical approach [25]. A preliminary codebook was created through both deductive analysis (informed by the interview guide and the Kirkpatrick framework) and inductive analysis. Codes were then defined as categories and sub-categories, and transcripts were iteratively reviewed to refine categories that captured breadth and nuance while eliminating redundancy. To ensure rigor, EM and AML coded one transcript in tandem, then independently coded two additional transcripts (10% of total) to reach agreement. Any discrepancies were resolved through discussion. The codebook can be viewed in Supplementary material 4.

## Results

### Participants

Members of five Australian HRECs in university, hospital, adult and paediatric settings were invited (four Queensland, one New South Wales) to participate. Of the 148 HREC members comprising the five invited HRECs, 24 subsequently consented within the recruitment timeframe (response rate 16.2%). An additional five genomics experts were invited to review the comprehensiveness and appropriateness of the content. During the recruitment timeframe, 374 unique visitors viewed the website, which housed the resource and other study-related information.

### Participant characteristics

Participant characteristics can be viewed in Table 1. Two thirds of interview participants identified as female (*n* = 20), and the majority (*n* = 27) were current or recent HREC members, including three of the five genomics experts (Table 1). The most reported HREC membership category was Category F (researcher) (*n* = 11), and all other membership categories were reported in three or more individuals, except for Category D (pastoral) (*n* = 1). As can be seen in Supplementary table 2, the proportion of each category is consistent with the compositions of the five invited HRECs, though research scientists are less represented (47% in HRECs and 36% in our study cohort). The majority of HREC members had been members for 2−5 years (*n* = 11), or 6−10 years (*n* = 8).

**Table 1:** Participant characteristics.

	*n*
**Total**	29
**Gender**	
Male	9
Female	20
**HREC Role**	
Category A - Chairperson	3
Category B – Lay member	5
Category C – Nurse or allied health professional	4
Category D – Pastoral member	1
Category E – Lawyer	3
Category F – Researcher	11
Not on a HREC	2
**Years of HREC Experience**	
One year or less	5
2−5 years	11
6−10 years	8
More than 10 years	3
Not on a HREC	2
**Genomics Experience**	
Yes	15
*Undertaken genomics education/professional experience*	13
*Genomics ethics training*	2
No	14
**Comfort reviewing genomics applications**	
Low	11
Moderate	11
High	5
Not on a HREC	2

Almost half of the participants (*n* = 15) had received some form of genomics training, of which 13 had genomics education/professional experience and two had completed a training course in genomics ethics. Few HREC members (*n* = 6) reported high levels of comfort reviewing genomics ethics applications, while the remaining participants reported either moderate (*n* = 10) or low (*n* = 11) confidence. Although not explicitly asked, six members of varying confidence levels (mostly low-to-moderate) volunteered that they rely on other HREC members when reviewing genomics ethics applications.

Interviews lasted 11−54 min (mean: 26 min, median: 24 min). Just over a quarter of participants (*n*= 8) reported not evaluating the entire content (e.g., not finishing videos), most of whom reported moderate to-high baseline confidence levels. Specifically, they reported already knowing the information from previous genomics education or professional experience. Participants who reviewed the resource in its entirety reported taking between 30 minutes and 2 h 45 min to go through the resource (mean: 1 h 20 min, median: 1 h 15 min). Of the participants that mentioned whether they reviewed the resource in a single or multiple sittings, most reviewed it over multiple sittings (*n* = 11/15).

### Evaluation of content and format (‘Reaction’ as per Kirkpatrick Model)

#### Content

Qualitative data and quotes relevant to content quality and volume can be seen in Table 2. When evaluating content, participants stated that the resource either met their expectations (*n* = 15), or they had no preconceived expectations to begin with (*n* = 9) (quotes 1 and 4). One participant reported expecting to see more comparisons of the ethical issues associated with genetic testing for different purposes (e.g. cancer/somatic testing) (quote 2), and another participant expected a more detailed explanation of the risks associated with the different genomics technologies (quote 3). Most participants, regardless of genomics background, found the content compatible with their current level of understanding (quote 5). Some participants with genomics knowledge felt that members without a genomics background may not understand the explanation of the types of genomics technologies. Four out of 14 participants with no prior genomics experience found the explanations of different genomic technologies “confusing” (quote 6). Additionally, most participants thought the resource addressed all the relevant topics required to understand and review a genomics ethics application (quote 7). Some participants from lay, legal, chair, and researcher categories (*n* = 5) believed that being on a HREC did not necessitate a detailed understanding of genomics theory, or the technologies used, but rather an awareness of the associated risks (quote 8).Participants spoke positively of the readability and diversity of presentation of content elements (quote 9) – in particular, participants appreciated the visual summaries (e.g., a table comparing ELSI risks between different genomic technologies) and a downloadable checklist outlining how to review a genomics ethics application (quote 10).

**Table 2:** Qualitative feedback: resource content and volume (as per Kirkpatrick “Reaction” element).

Category	Sub-category			Illustrative Quotes^a^
Content quality	Expected content		Was present	*“[…] I was expecting to see a Genomics 101 [module] and […] something about return of results and I did. And then I was expecting to see ELSI explained to me. And then I was a hundred percent expecting to see reviewing a genomics application.”* Lana* (research member & genomics expert) (quote 1)
			Was not present	*“I was expecting it to be divided into germline, somatic, circulating tumour, like that, those sorts of different things. Um, because they’ve got implications for the ethical issues. And I felt like that was not really teased out in it. Like I felt like a lot of it focused on germline, which makes sense. But you do need to make it clear […].”* Mindy* (research member & genomics expert) (quote 2) *“[It] says […] this technology is low risk and this technology is high risk, but I actually didn’t know what [it] meant by risk at all.”* Melanie* (research member, genomics experience) (quote 3)
			Had no expectations	*“I just came in knowing that I just knew so little about it, so I didn’t really know what I didn’t know.”* Bridget (counsellor/allied health member, no genomics experience) (quote 4)
	Ease of readability		Compatible with current level of understanding	*“[…] it was a really good level for people who are naive because I understood it and I have nothing more than your basic, you know, year 12 science […] I thought it would be scarier and it wasn’t.”* Stella* (research member, no genomics experience) (quote 5)
			Not compatible with current level of understanding	*“I was a little bit confused in some of the technologies […] I don’t feel like I’ve got a full understanding still. So I think I needed a little bit more info there.”* Melanie* (research member, genomics experience) (quote 6)
	Topics covered		Appropriate	*“[…]what I wanted to know specifically was addressed in the last lesson. Um, but now that I’ve done the course, I needed all the previous lessons to fully understand that one.”* Elisa* (research member, no genomics experience) (quote 7)
			Less interested or less relevant	*“I think as a lay person, I just need to understand the risk more than, than how it works.”* Lesley* (lawyer, no genomics experience) (quote 8)
	Positive feedback on content		General content feedback	*“I was very appreciative of this course because I think I didn’t fully understand the ethical implications from the tests and the differences between them. And yeah, to be told explicitly is just wonderful. I really enjoyed that.”* Elisa* (research member, no genomics experience) (quote 9)
			Figures and tables	*“Love the checklist. Honestly, I think that’s such a helpful part of it […]”* Elisa* (research member, no genomics experience) (quote 10)
			Videos	*“[…] one of the really good elements of all the five modules was that […] introductory five to seven minute video”* Isaac* (lay member, no genomics experience) (quote 11) *“It was good how you had [patient] the whole way through because it did make it, um, sort of engaging and [good for] following along.”* Stella* (research member, no genomics experience) (quote 12)
Content quantity	Contained right amount of content			*“I think the volume was good. I think, um, any less then you wouldn’t be able to like brush [up] on important topics and any more it would become quite a big task then for people. I think that it, you’ve sat in the Goldilocks zone quite comfortably there.”* Stacey* (genomics expert) (quote 13)
	Appropriate amount of time to spend going through resource			*“I think one to two hours is a reasonable amount of time.”* Bill* (research member & genetics expert) (quote 14)
Recommendations	Add or expand content	Familial and community risk		*“[…] there’s a, a big focus on individual risk. But there’s also, and it is addressed later in the module, but less clearly I suppose, is the potential risk to familial and community […] especially when considering minority communities or First Nations communities […].”* Stacey* (genomics expert) (quote 15)
		*Research considerations for diverse populations*		*“But with your community, with your ethnic group, with like lots of different areas. Um, and so there are group harms that come from that. […] researchers need to have a plan for dealing with and should be reflected in the ethics application. […] In Australia, you really need to have a specific mention of Aboriginal and Torres Strait Islander concerns. […] those same principles apply for everyone, but there’s other underrepresented groups in genomics, so lots of migrant communities.”* Mindy* (research member & genomics expert) (quote 16)
		Data storage and secondary access to data		*“[…] how this data will be stored, where, who’s gonna have access to it long term, whether this will be stored on a cloud, how secure is the cloud, whether it’s gonna be stored on a hard drive […] I don’t think there was a lot of comments on that.”* Stacey* (genomics expert) (quote 17) *“ […] we should be highlighting in the HREA consent question about the type of consent being requested, whether it’s specific, extended or unspecified, because it obviously pertains to the future use of the data.”* Rita* (lay member, no genomics experience) (quote 18)
		Genomics Technologies ELSI Summary Table		*“ [with regards to the risk summary table, are there] risks of the technology being wrong, risks of the technology identifying incidental findings, risks of the technology, all the different risks. […] I think in some ways I would like to see it unpacked.”* Sherri* (genomics expert) (quote 19)
		Optional additional resources		*[pertaining to Genomics 101 module]” I found myself diving into Google to answer questions that I had […]I would’ve preferred more […] but I can’t say that that would necessarily be the case with all HREC members. And maybe there’s a […] compromise to be drawn there in perhaps being able to lead people off to more information should they find that they would like it.”* Rita* (lay member, no genomics experience) (quote 20)
		More direction for reviewing genomics applications		*“I did wonder whether with the checklist there could be also hints. So, you know, if the checklist says […] check for this or look, look for this, what is the optimum? What, what are we looking for?”* Margo* (research member, genomics experience) (quote 21)
	Plain English			*“I was thinking [what] would be helpful would be a glossary that you could refer to […] there’s quite a lot of acronyms and some of the acronyms are ones that mean different things to me clinically.”* Bridget* (counsellor/allied health member, no genomics experience) (quote 22)
	Accessibility			*“It’s not terribly accessible at the moment. […] your captions, your transcripts, your ways in, um, your contrasts, those kinds of things.”* Sherri* (genomics expert) (quote 23)

^a^Numbered for ease of in-text referencing.

*pseudonym.

Overall, participants believed the resource contained an ideal volume of content (Table 2, quote 13). When asked what they thought was an appropriate amount of time to spend upskilling in this area, most answers ranged between 1-2 hours (quote 14).

Participants recommended additional detail on specific topics, including risks pertaining to the family and community (particularly diverse populations e.g Indigenous Australians) (quotes 15-16), storage of and secondary access to genomics data (quotes 17-18), ELSI Summary table (quote 19), additional resources regarding genomics basics (quote 20), and more directivity/examples for reviewing a genomics ethics application (quote 21). Other suggestions included a glossary and integrating more accessibility features (quotes 22-23). The extent to which each of these recommendations align with HELF principles is reviewed in the discussion section.

#### Format and delivery

Qualitative data relevant to delivery, format, and accessibility can be seen in Table 3. Most participants (*n* = 25) found the resource easy to access and navigate (quotes 24-25), although some reported feeling lost at times as there was no explicit numbering or ordering of the modules (quote 26). Participants enjoyed the multimodal nature of the resource, which they found to be engaging and catered to different learning styles (quote 27). Many appreciated the repetition of the content across both written and video contexts served to consolidate the information (quote 28). The organisation and order of the modules was positively regarded as having a logical flow and following a ‘top-down’ approach (quote 29), but participants also appreciated the flexible and asynchronous nature of the resource, with the ability to personalise their journey and skip between modules based on their pre-existing knowledge, as opposed to needing to complete the modules in a pre-ordained order (quote 30). However, one participant noted they found this feature distracting and would prematurely skip to modules that looked interesting (quote 31).

**Table 3:** Qualitative feedback: resource delivery and format (as per Kirkpatrick “Reaction” element).

Category	Sub-Category		Illustrative Quotes^a^
Navigation	Ease of access		*“I would know also that if I needed a refresher, I could easily go back to the link. I know where it is now to be found.”* Isaac^b^ (lay member, no genomics experience) (quote 24)
	Ease of navigation	Easy to navigate	*“I’ve seen a lot of educational modules that are […] just a nightmare to navigate, and this was nice. Not too many pages, not too much clicking.”* Mindy^b^ (research member & genomics expert) (quote 25)
		Unclear navigation	*“[…] I got lost. What, having gone through the whole course, I quite frequently said, where am I in the course? Is this the third module popping up out of five?”* Isaac^b^ (lay member, no genomics experience) (quote 26)
Website layout & format	What works well	Multimodal	*“[…] it was a great blend of written and visual materials to cater to different learning styles […]”* Stacey^b^ (genomics expert) (quote 27)
		Good use of repetition	*“I needed to have that reiterated to me, um, just because I feel like the majority of people don’t have a lot of experience in this area. So hearing it in two different ways helped. And then also then having the written consolidation afterwards I thought was good.”* Elisa^b^ (research member, no genomics experience) (quote 28)
		Organisation & order of modules	*“It had a good flow to it, so it all sort of fed into each other logically, which made it interesting and easy to keep up with as you went through the modules.[…] It’s like a natural progression through from like a really good intro and it kind of gets more and more narrow to then what I need to know practically. ”* Stacey^b^ (genomics expert) (quote 29)
		Asynchronous and customisable learning experience	*“I was able to come in here as a fairly experienced person and jump straight to something that was new to me.”* Lana* (research member & genomics expert) (quote 28) *“I think anyone could do it, you know, even if they were really a busy clinician or a busy lay person, they could easily get the modules done at a pace that was convenient for them.”* Marlin^b^ (counsellor/allied health member, genomics experience) (quote 30)
	What didn’t work well	Not a structured, linear course	*“[…] I was clicking on ones that interested me. I actually jumped to module three or four, you know, initially… I didn’t do it in sequential order […]”* Brian^b^ (chairperson, genomics experience) (quote 31)
Recommendations	Formatting and layout	Clearer and easier navigation	*“[…] your navigation at the moment is letting you down […] The, the fact that I only just found your slider [navigation bar] […] possibly means that, uh, that, that, that someone else may not find it and then potentially get frustrated as they’re taken through.”* Sherri^b^ (genomics expert) (quote 32)
		Estimated read time per page	*“[…] you can say estimated read time.”* Lana^b^ (research member & genomics expert) (quote 33)
		Number the modules & pages	*“[…] name them, label them, because I got lost. What, having gone through the whole course, I quite frequently said, where am I in the course? Uh, is this the third module popping up out of five?”* Isaac^b^ (lay member, no genomics experience) (quote 34)
		Progress bar per module and course	*“So you’ve actually got a progress bar right at the very beginning. ‘Cause I now go back to it said I’ve completed a hundred percent, but you need, I think, a progress bar for each module.”* Issac^b^ (lay member, no genomics experience) (quote 35)
		Search function or index	*“Maybe some kind of index that you could jump to for the future would be good. So say if you had an application and you really just, you don’t want to go through the whole course again, you just want to jump to a particular place and just review that.”* Noreen^b^ (research member, no genomics experience) (quote 36)
		Choose from structured or non-structured delivery	*“[…] pedagogical approach, […] could be sequential learning so that you’re, you’re building upon the knowledge that you want to give.”* Brian^b^ (chairperson, genomics experience) (quote 37)
	Active Learning and Engagement	Reflective Questions	*“[…] pose a question, like, from what we’ve covered thus far in the course, what would might you be thinking would be potential, uh, areas of, of conflict or challenge, or what sort of questions would be coming up in your mind?”* Isaac^b^ (lay member, no genomics experience) (quote 38)
		‘Check your knowledge’ activities	*“[…]the idea of being able to use some kind of adaptive learning or some kind of quizzing, knowledge check, feedback, drag and drop, um, instant check, I think is still potentially viable to offer in a thing that’s for adults and professional adults.”* Sherri^b^ (genomics expert) (quote 39)
		Case study and examples	*“I think examples and case studies sort of articulating why some types of practise are, what we should be looking for […] And sort of saying, well, this would be the ideal situation […] like giving them examples of what they could ask for from researchers.”* Mindy^b^ (research member & genomics expert) (quote 40)
	Additional features	Downloadable summaries	*“[…]that written text that could be a single document that somebody can download and have as a reference. Um, yeah, I think particularly some people that are less sort of tech savvy or more inclined to use hard copies of things, but that would be useful for them to refer back to […]”* Margo^b^ (research member, genomics experience) (quote 41)
		Completion page/certificate	*“[…] there’s nothing for me to say, hooray, I’ve done something.[…] for some people this would be a handy thing to have a certificate to get some credit points for.”* Lana^b^ (research member & genomics expert) (quote 42)
		Real-time learning opportunities	*“a live webinar, just because […] you basically have to engage properly.”* Arthur^b^ (legal member, genomics experience) (quote 43) *“potentially the opportunity to email members of your team for specific questions [about genomics ethics applications] or updates”* Brian^b^ (chairperson, genomics experience) (quote 44)

^a^Numbered for ease of in-text referencing.

^b^pseudonym.

Participants suggested the inclusion of a clearly visible navigation menu, together with time estimates per module, numbering modules/pages, a progress bar, and search function/index (quotes 32-36). Additional suggested features included integrating active learning through interactive elements, as well as downloadable summaries, a completion page and downloadable certificate at the end of the resource, customisable resource delivery (login system and option of structured or unstructured delivery), and real-time learning opportunities such as webinars or email hotlines (quotes 37-44). The extent to which each of these recommendations align with HELF principles is reviewed in the discussion section.

### Utility (‘Learning’ as per Kirkpatrick Model)

Qualitative data and quotes relevant to perceived resource utility can be seen in Table 4. Over three-quarters of participants (*n* = 23) stated they would refer to this resource if, and when they receive a genomics ethics application to review in the future (quote 45-47). All HREC members that reported low/moderate baseline confidence (*n* = 21) reported that this resource had improved their confidence reviewing and discussing genomics ethics applications (quote 48). Furthermore, three of the six HREC members who self-described as very confident at baseline also reported improvements in genomic confidence. Most participants would recommend this resource to other HREC members (*n* = 28). Furthermore, some participants (*n* = 7) volunteered that they would recommend the resource to researchers (quote 49), to guide the design and development of ethically defensible applications or enable them to share videos with participants to enhance understanding of basic genetics concepts.

**Table 4:** Qualitative feedback: perceived resource utility (as per Kirkpatrick “Learning”).

Category	Illustrative Quotes^a^
Useful as a reference resource	*“[…] even if I don’t carry it in my head, I would go back when I got a genomic study and I would look at it again and work through it again. So I think it was very useful in that regard.”* Lesley^b^ (lawyer, no genomics experience) (quote 45) *“[…] we don’t see [genomics] applications all the time [in our HREC]. They come in sporadically and […] if they can have a […] resource that they can go and relearn a particular aspect or search for knowledge on a particular area, I think that that would be very, very powerful.”* Brian^b^ (chairperson, genomics experience) (quote 46)
Would use as needed	*“I wouldn’t do it proactively to upskill in case I get a review. I’d be coming here once I’ve got a review.”* Lana^b^ (research member & genomics expert) (quote 47)
Improved confidence reviewing HREC applications	*“I feel much more confident about what to look for in the application and just generally education around the types of different technologies and the risks associated with the data that’s generated from that…”* Noreen^b^ (research member, no genomics experience) (quote 48)
Utility outside of HRECs	*“I think it gives people, um, not just people that are viewing ethics applications, but also people that are actually embarking on this type of research, an idea of what an optimum consenting process looks like.”* Margo^b^ (research member, genomics experience) (quote 49)

^a^Numbered for ease of in-text referencing.

^b^pseudonym.

## Discussion

This study qualitatively evaluated the acceptability and utility of a new online resource designed to empower HREC members to review genomics applications. All participants found the resource easy to access, and most reported ease of navigation, clear layout and formatting, and improved confidence in their ability to review genomics applications, thereby addressing the ‘reaction’ and ‘learning’ elements of the Kirkpatrick evaluation of an educational resource. Participants provided constructive feedback to improve content, navigation, learning design, and evidence of completion, which align with the HELF principles.

Participants largely appreciated the flexibility in website navigation, which allowed them to select and skip modules according to their specific learning needs and prior knowledge. One suggested feature was the option for structured delivery, in which users progress through the resource in a linear fashion, course-like manner, and cannot ‘skip ahead’. Prior research has shown that learners without any prior knowledge demonstrate increased recollection when a course is presented in a structured fashion, while learners with prior knowledge benefit more from a more ‘à la carte’ course progression [26]. Therefore, ideally users should be able to choose between structured and non-structured resource delivery to suit their learning needs. Additionally, the flexibility and convenience provided by an asynchronous online courses suits adult learners who are typically required to balance their education with other duties, such as employment or caring responsibilities [27]. However, some participants requested additional, real-time learning opportunities such as webinars or email hotlines, serving a dual-purpose of absorbing content in a synchronous manner and having access to an expert if they have specific questions about an application they are reviewing. While synchronous learning does not consistently improve learning outcomes [28], some learners perceive greater learning gains and enjoyment in synchronous rather than asynchronous learning [29]. The ability to move freely between modules promotes autonomy, which is an essential component of the HELF principles ‘emotions and learning’ and ‘learning to learn and higher order thinking.’ [21] Autonomy empowers the learner to personalise consumption, which increases satisfaction with the learning experience, and promotes continuous self-evaluation of knowledge and learning [21]. Both the positive participant reactions to the resource, and the perceived improved confidence in skills align well with successful evaluation relative to Levels 1 and 2 of the Kirkpatrick Model [24].

A clear, visible navigation bar and search function would enhance custom navigation and allow participants to easily access the desired content, thereby reducing cognitive load. Cognitive load refers to the amount of information one’s working memory can process at a given time, and extraneous cognitive load indicates the utilisation of mental resources for elements which do not contribute to learning [30]. Unclear navigation and a lack of an ability to quickly find information both increase extraneous cognitive load, thereby intensifying the risk of cognitive overload [30], which has been shown to hinder learning and retention in adults [31, 32]. Participants suggested time estimates for completing each module, which has been shown to empower learners to manage their time, reduces attrition, and improve outcomes in higher education online courses [33, 34]. Similarly, progress bars, a learning approach adapted from videogames (gamification) [35], can improve time management and positively influence motivation and completion rates in adult learners [36, 37]. Participants requested that a completion page/certificate be included in the next iteration. Rewards and incentives, such as completion certificates, have been shown to decrease attrition and motivate successful completion in adult and online learning [38]. Completion certificates have been utilised by HRECs in Australia to monitor compliance with Good Clinical Practice training [39], and thus completion certificates for this resource could possibly be used to demonstrate that a significant portion of HREC members are capable in this area, though it will be up to individual HREC chairs to decide whether this is a requirement.

Participants suggested that more active learning and interactive elements would enhance the learning experience. It is well-established in the education field that incorporating active learning elements improves learning outcomes by encouraging learners to engage with the material through thinking, investigating, problem solving, and creating [40]. Specifically, it allows learners to develop and refine their conceptual understanding of a topic across multiple contexts, and in doing so promotes deeper and more meaningful learning [41]. In adult learning specifically, the integration of active learning elements has consistently been associated with increased critical thinking skills, motivation, and retention in both face-to-face [42] and online settings [43, 44]. Therefore, the integration of active learning elements such as interactive ‘check your knowledge’ activities, case studies, and reflective questions can be utilised to maximise learning outcomes. All these elements speak, directly or indirectly, to the HELF principle of ‘emotions and learning’, which increases motivation, sense of accomplishment and promotes opportunities for cognitive skill utilisation such as problem-solving and critical thinking [21]. Additionally, interactive elements facilitate active learning, which pertains to ‘contextual learning’ i.e., developing capability to apply the information to novel or atypical contexts [21, 45]. Unfortunately, the Squarespace platform does not support these learning features, and thus, as per the navigation limitations noted above, the next iteration will necessitate an alternative online platform.

Most participants expressed positive feedback regarding the quality and quantity of the content, including the appropriateness of the topics covered, ease of readability and the time taken to complete being deemed ‘appropriate’. Of note, many participants appreciated the utility of the table comparing the ELSI risks relative to different genomic technologies. While a comparison of the different genomic technologies utilised in research is not a novel concept, comparisons typically highlight differences in mechanisms, cost-effectiveness, resolution, sample type or coverage [46–48]. To our knowledge, this is the first comparison of ELSI risk relative to genomic technologies. Additional suggested content pertained to genomic research involving Aboriginal and Torres Strait Islander Peoples. Considerations include respecting the authority of the community, partnering with community members in the study design and research processes, developing resources for consenting participants of culturally diverse backgrounds, and regularly reviewing research processes and findings with community (if applicable) [49, 50]. These considerations and practices are relevant for any genomics research involving underrepresented populations [51]. Thus, this will be explored in more depth and detail in the future iterations of the educational resource. Furthermore, a modified version of the website could be created for other countries (e.g., IRBs in North America) reflecting their respective national guidelines and ancestral compositions.

The primary goal of this educational resource is to improve the quality of genomic research. The feedback was positive in terms of acceptability, feasibility and utility, the ease of navigation and re-accessing, and improvements in confidence. Therefore, we believe that the revised iteration has the potential to empower HREC members to understand the nuanced risks and benefits for individual genomics research projects and highlight areas for further reflection in the formation of an ethically defensible plan.

### Limitations

Despite having a study cohort composition consistent with the composition of the five participating HRECs, we had hoped to attract more lay/legal/pastoral members as they were less likely to be genomically literate. In future studies, targeted recruitment of lay/legal/pastoral HREC members is needed to better understand their perspectives, experiences, and the utility of the resource for non-medical/scientific members. Additionally, only HRECs from two Australian states were invited to participate and thus there may be state-specific processes or perspectives which were not captured.

## Conclusions

This is the first study to develop and evaluate a genomic educational resource for ethics reviews committees, specifically Australian HRECs particularly. Participants reported ease of accessing and navigating the resource, an appropriate quantity of information which can be reviewed in a reasonable time, and the novel inclusion of ELSI considerations specific to genomics technologies. Areas for improvement pertained to content, navigation, learning design, and evidence of completion. The next iteration will be quantitatively evaluated both short- and long-term (2−3 years) with a larger, national sample to quantitatively determine the resource’s utility and efficacy in increasing HREC member genomics knowledge and confidence. Bespoke adaptation could generate resources to enhance training of ethics committees in other countries and to empower researchers to prepare quality HREC applications.

## Supplementary information

Supplementary Materials

Supplementary Table 1

Supplementary Table 2

## Data Availability

There is no other relevant data from this manuscript.
