# Supplementary material for: Empowering human research ethics committees to review genomics applications: evaluating the utility of a custom online education resource
Source: Eur J Hum Genet. 2025 Apr 17;33(7):945–55. doi: 10.1038/s41431-025-01846-5 (PMC12229321; doi:10.1038/s41431-025-01846-5)
Supplement: Supplementary file 1 — Supplementary Materials [file 41431_2025_1846_MOESM1_ESM.docx]

# Supplementary material 1: Mapping resource elements to Higher Education Learning Framework (HELF)^22^

| **HELF Principle** | **Relevant elements in educational resource** |
| --- | --- |
| **Learning as becoming** | - HREC members recognize their roles as lifelong learners and are committed to continuous learning, understanding how this educational resource aligns with their role |
| **Contextual learning** | - Resource contextualizes the ethical considerations for genomics research from the National Statement into real-world scenarios to promote deeper and more meaningful learning - E.g. Videos simulating a patient being consented to a research study, a figure outlining the ethical risks of different types of genomic technologies, and a checklist outlining key considerations when reviewing a genomics ethics application |
| **Learning to learn and higher order thinking** | - Resource design encouraged learner autonomy and allowed them to self-assess and identify knowledge gaps, thereby promoting engagement, more positive learner attitudes, and allowing choice in their learning - The resource was unstructured and non-linear in its delivery, which allowed learners to skip to and between modules depending on their prior knowledge and learning needs - The multimodal nature of the resource (including text, live action, and animated videos) increased autonomy by allowing learners to choose how content is consumed (as the content is consistent between the videos and text) |
| **Emotions and learning** | - Emotions play a key role in promoting engagement, positive learner attitudes, behaviours and retention. Creating a relationship with learners creates a positive disposition toward learning and increases the motivation to engage with the content - In lieu of real-time social interaction, a front-page video of AML introducing themselves and the course was incorporated to establish a relationship with learner. This relationship was maintained by including their face and voiceover in the introductory videos of each module - As mentioned above, allowing users to choose how they consume the content of the resource promotes autonomy, thereby increasing satisfaction with the learning process - Learner engagement was fostered through elements such as ‘did you know?’ bubbles, interactive drop-down accordions, external links to Youtube videos, and further reading |
| **Deep and meaningful learning** | - Deep and meaningful learning represents the optimal goal of any educational experience, and involves encouraging learners to actively engage with course materials - This principle has broad applications within an educational environment and is reflected in the principles outlined previously. It is through the practical application of these principles that learners can truly experience deep and meaningful learning |
| **Interactive Learning** | - Pertains to enhancing learning experience through social dynamics - Less relevant to this resource as it is an online, asynchronous resource |
| **Learning challenge and difficulty** | - Pertains to promoting greater depth of understanding by evoking and resolving struggle through effort - Less relevant to this resource as it is a short course designed to be a comprehensive guide that users can learn from and refer back to |

# Supplementary material 2: Screenshots from HREC resource

##
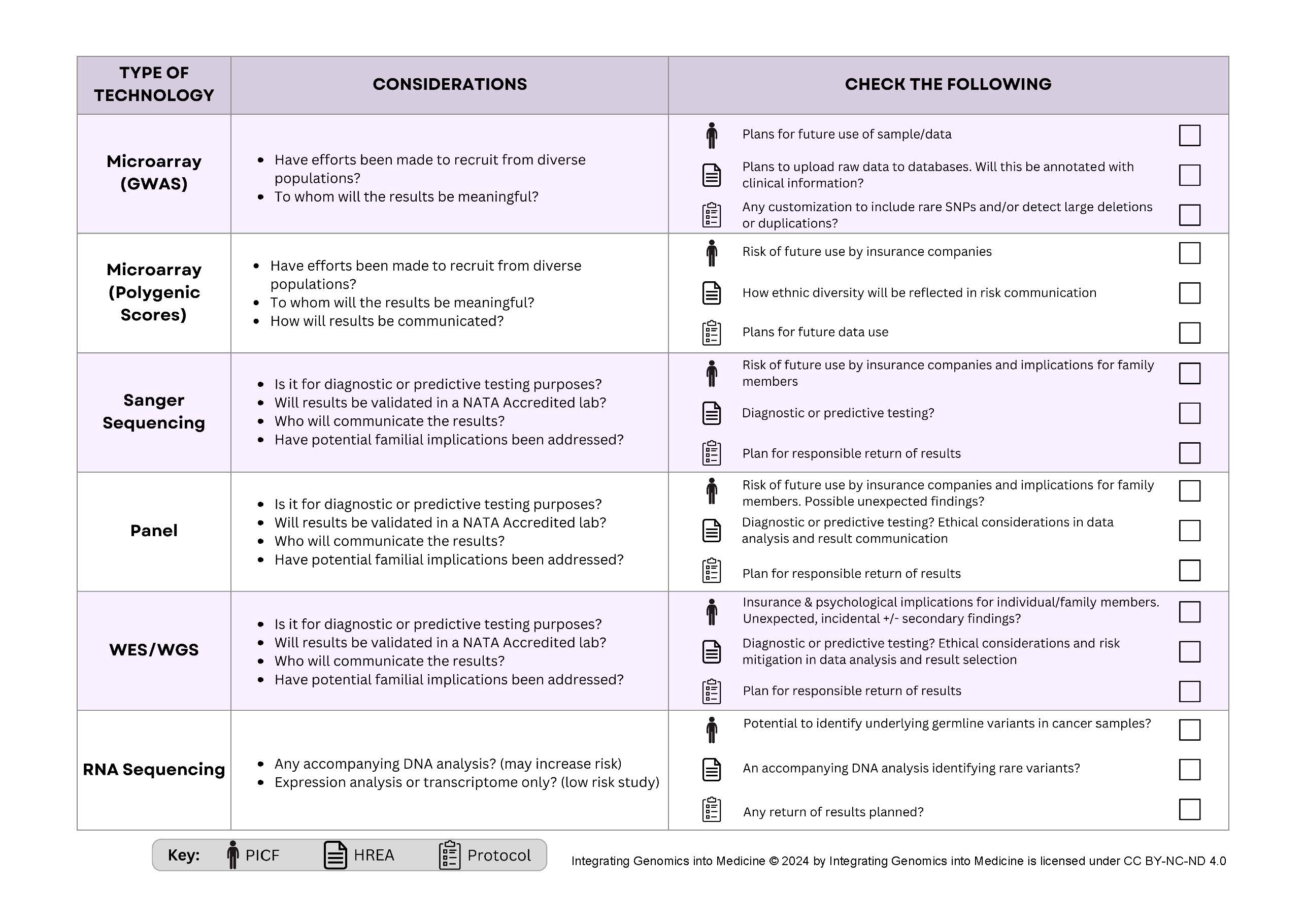
Supplementary material 2a: Checklist for reviewing genomics ethics applications

##
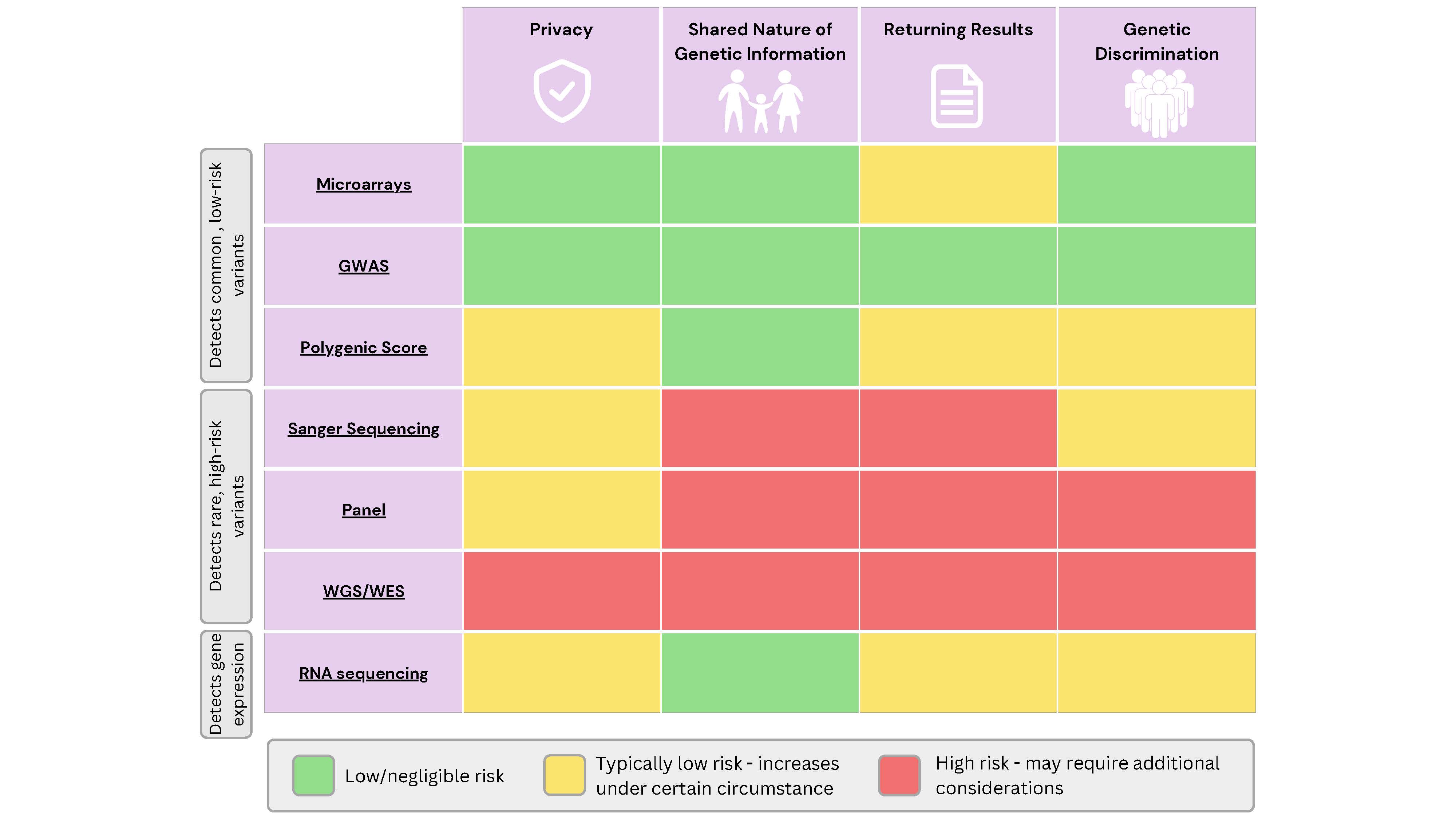
Supplementary material 2b: Summary of ELSI risk according to genomics technology used

## Supplementary material 2c: Screenshot showing text elements and drop-down accordions


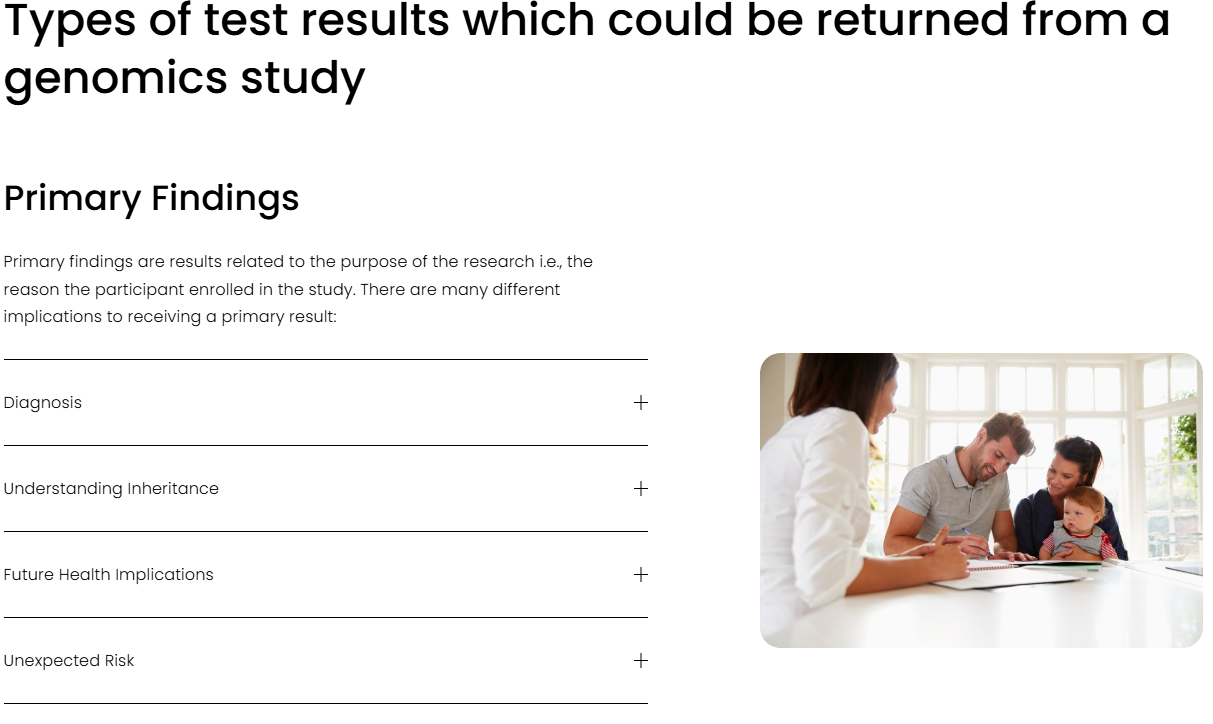

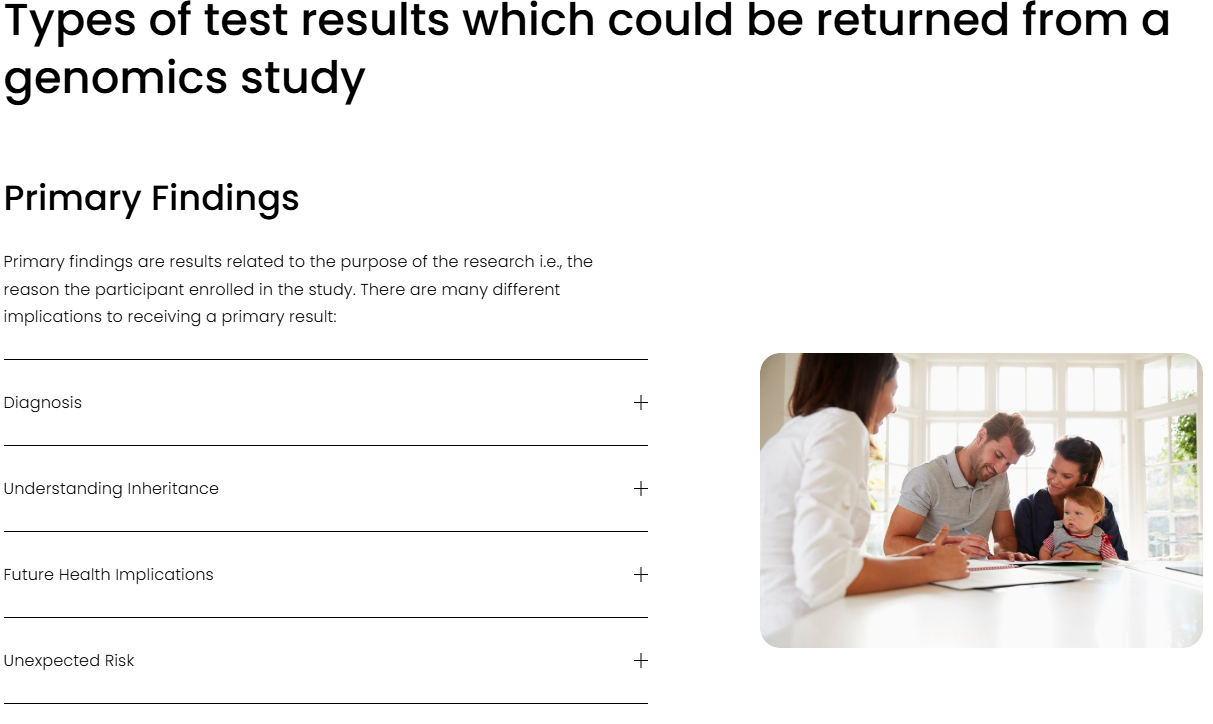

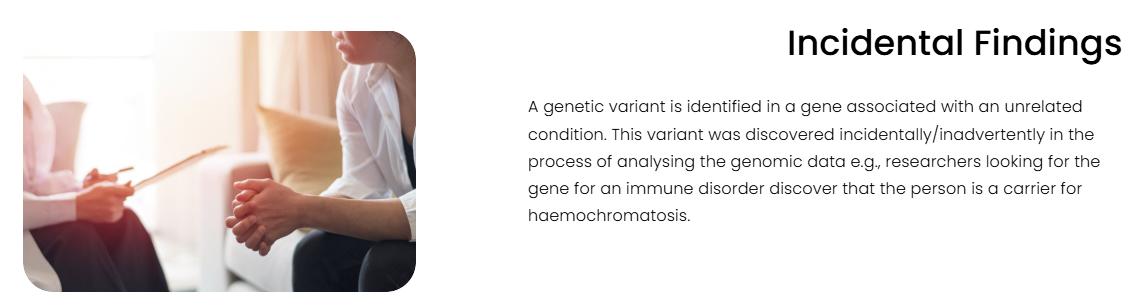

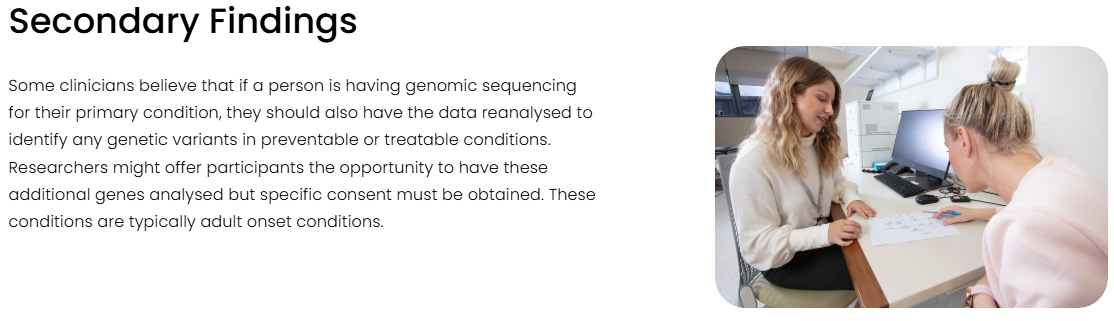


## Supplementary material 2d: Screenshot showing lecture style videos, figures, and text elements


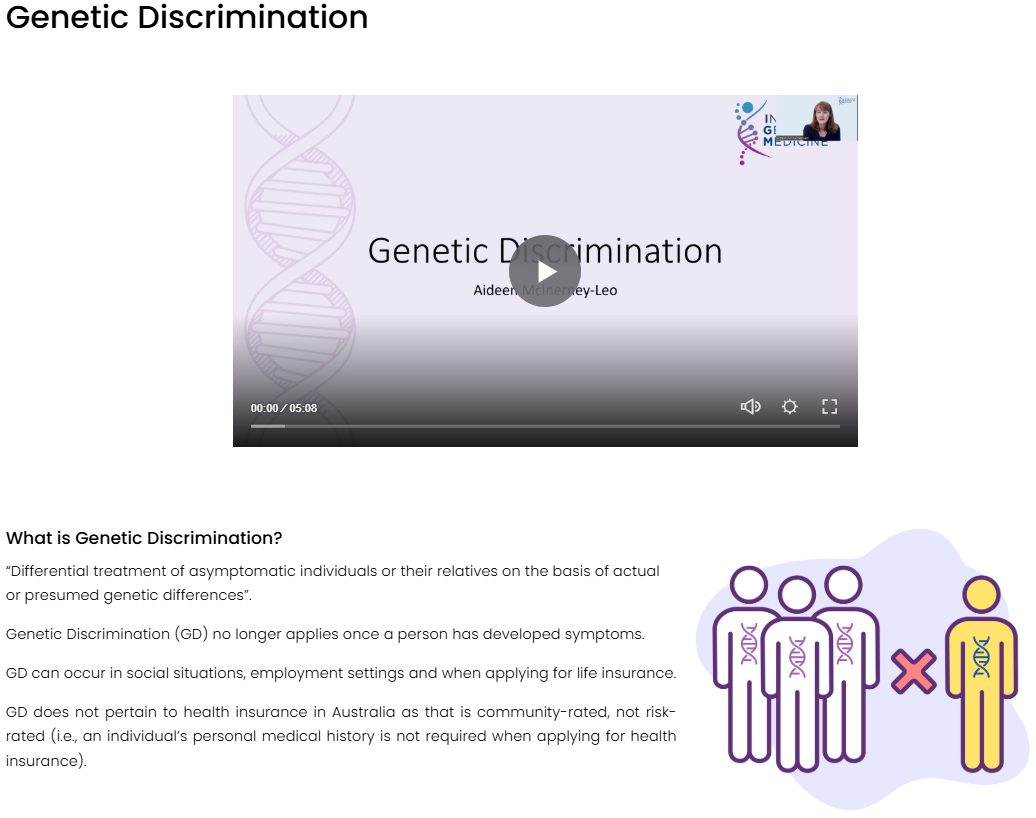

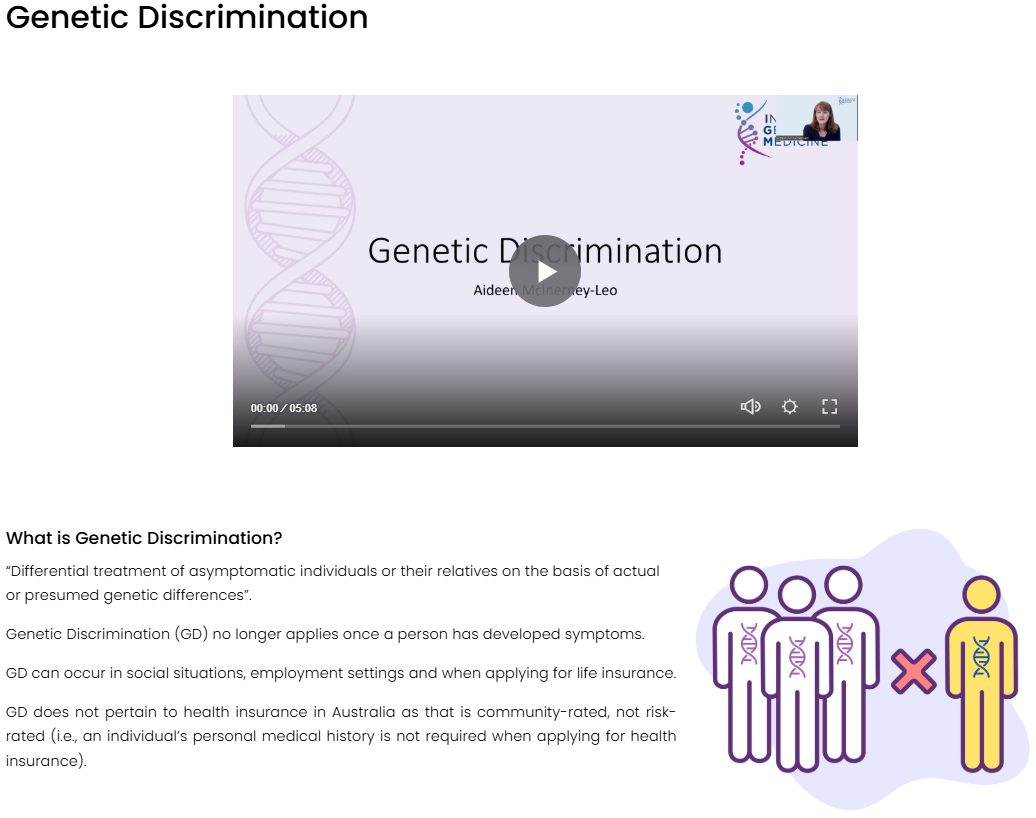

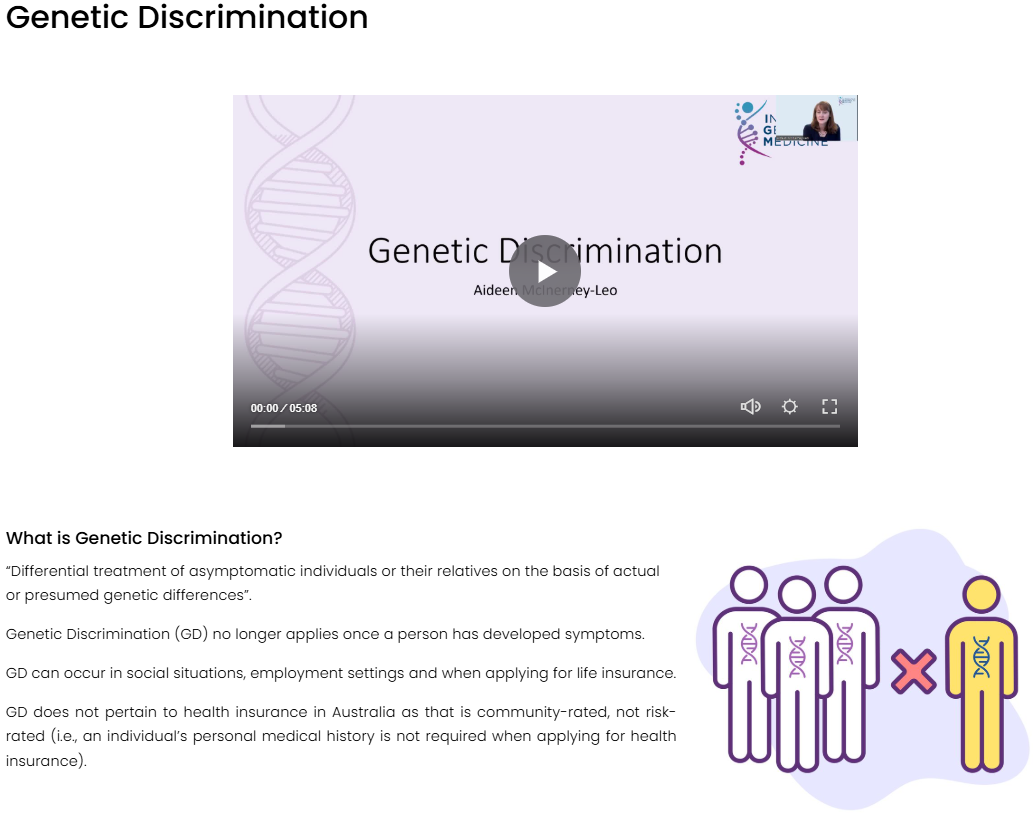


# Supplementary material 3: Semi-structured Interview Guide

**Introductory script**

- Thank participant for agreeing to take part in this study.
- Remind the participant that the purpose of this interview is to discuss their experiences with reviewing ethics applications with genomics, and their thoughts on the educational resource.
- Discuss the role of the interviewer: to raise topics for discussion and then to listen as the participant shares their views and experiences.
- Reassure the participant that they are free to talk about any aspect of their experience or attitudes. There are no right or wrong or even typical answers to any of the questions that we will discuss.
- Reassure confidentiality and the participant’s right to stop the interview at any time.
- Clarify that the interview will take approximately 15-20 minutes (even though participant information form said 30-60 minutes).
- Highlight that we may use the terms ‘genomics’ and ‘genetics’ interchangeably.
- Remind the participant that, with their permission, the interview will be recorded.
- Ask whether the participant has any questions before we begin.

*Start recording*

**Experience with reviewing human research ethics applications**

1. For our records could you please tell me a little more about you?
   1. Which gender do you identify with?
   2. What role/category you serve on HREC?
   3. How many years have you served on a HREC?
   4. What is your professional background?
2. Can you tell me about your experience as a reviewer with a HREC?
   1. Are there certain types of applications with which you feel more/less comfortable?
3. What is your experience reviewing ethical proposals that include genomics?
   1. How comfortable do you feel reviewing genomics applications?
   2. How comfortable do you feel participating in discussions regarding genomics applications?
4. Can you tell me about your background level of understanding of genomics?
   1. Have you had any formal training or specific experience in the area?

**Format/navigation of the resource**

1. How easy was it to access the resource?
2. How easy was it to navigate the resource?
3. Was the modular system helpful?
4. Before starting to use the resource, were there specific topics or areas you were interested in learning about?
5. Did you find it easy to find these specific topics and/or was it easy to find answers to specific questions?

**Nature of the content**

1. How did using the resource make you feel?
   1. Did you find any sections particularly useful? Which ones and why?
   2. Did you find any sections to be unhelpful? Which ones and why?
2. Was the resource appropriate for your level of understanding?
   1. Were any of the resources confusing?
3. In the first module, Genomics 101, did you find the two Youtube videos from the Garvan Institute useful?

**Volume of the content**

1. How much time did you spend on the resource?
   1. Did this seem like a reasonable/feasible amount of time?
   2. What would be the ideal amount of time to spend upskilling in this area?
2. Would you have preferred more or less content? Can you explain why?

**Opportunities to improve the resource**

1. Do you think this resource has improved your confidence in reviewing/discussing ethics applications that include genomics?
2. After reviewing the materials do you feel more likely to engage in reviewing ethics applications that include genomics?
3. Would you recommend this resource to other HREC members?
   1. Why?
   2. Do you think they would be helpful to particular HREC member types?

**Overall recommendations**

1. Do you have any overall comments regarding what you think works well in the current website?
2. Do you have any overall comments about how we could improve the website?
3. Are there any specific sections that would benefit from changes?
   1. Which ones and why?
   2. What would you recommend?

**Interview debriefing and closure script**

- ‘I have no further questions. Is there anything I didn’t ask about that you would like to mention?’
- Thank the participant for sharing their experiences.
- Ask how the participant felt about taking part in the interview.
- Do they still have the telephone number in case they would like to contact us in the future?

# Supplementary material 4: Codebook

## HREC Interviews Coding Tree

1. Interviewee characteristics
   1. Gender
      1. Male
      2. Female
   2. Role on HREC
      1. Category A - Chairperson
      2. Category B – Lay member
      3. Category C – Counsellor/Nurse/Allied health professional
      4. Category D – Pastoral/spiritual leader
      5. Category E – Lawyer
      6. Category F – Research experience
      7. Not on a HREC
   3. HREC Experience
      1. Number of years
      2. University HREC
      3. Hospital HREC
      4. Experience preparing ethics applications
   4. Experience with genomics ethics applications
      1. Comfort reviewing genomics applications
         1. Low
         2. Moderate
         3. High
         4. Relies heavily on other HREC members
      2. Genomic confidence
         1. Low
         2. High
   5. Genomics Experience
      1. No
      2. Yes
         1. Genomics Training
            1. Qualification in genomics
         2. Ethics Training
         3. Professional exposure
   6. Motivation to participate in study
2. Delivery
   1. Navigation
      1. Ease of Access
      2. Ease of Navigation
         1. Easy to navigate
         2. Unclear navigation
   2. Website layout/format
      1. What works well
         1. Multimodal
         2. Asynchronous and customizable learning experience (pacing and skipping content)
         3. Good use of repetition
         4. Organization and order of modules
      2. What doesn’t work well
         1. Non-structured learning
3. Content
   1. Content quality
      1. Expected content
         1. Was present
         2. Was not present
         3. No prior expectations
      2. Ease of readability
         1. Compatible with current level of understanding
         2. Not compatible with current level of understanding
            1. Predicting ‘lay member’ understanding
      3. Topics covered
         1. Appropriate
         2. Less interested/less relevant
      4. Positive feedback on content
         1. General feedback
         2. Figures, diagrams and tables
         3. Videos
            1. Garvan videos
            2. Custom videos
   2. Content quantity
      1. Volume of content
         1. Too much
         2. Too little
         3. Right amount
      2. Time taken
         1. Amount of time
         2. Appropriate amount of time
         3. Predicting time it would take for others
         4. Whether reviewed in one sitting or interrupted
      3. Did not cover all elements
         1. Didn’t watch all video to end
         2. Already knew content
   3. Utility of resource
      1. Useful to refer back to
      2. Utility outside of HRECs
      3. Would use only on a needs basis
4. Recommendations
   1. Content
      1. Use plain English
         1. Nomenclature
         2. Glossary
         3. Avoid jargon
      2. Add or expand content
         1. Distinguish between clinical and research
         2. Genomics technology ELSI summary
         3. Data storage and secondary access to data
         4. Familial and community risk
            1. Research considerations for diverse populations
         5. More directivity in ‘reviewing applications’ section
            1. Checklist for reviewing genomics ethics applications
         6. Role-play scenarios
         7. Additional resources
         8. Contextualize content in terms of National Statement
      3. Remove content
      4. Discrepancies and typos
   2. Active learning and engagement
      1. Pose questions to learner then answer
      2. Check your knowledge questions or activities
         1. Concerns and alternative opinions
      3. FAQ section
      4. Case study and examples
         1. HREC roleplay scenario
      5. More ‘did you know’ facts
   3. Formatting and layout
      1. Improved navigation
         1. Personalize navigation experience
         2. Progress bar for modules
         3. Estimate read time per page
         4. Number modules & pages
         5. Clearer navigation menu
         6. Index or search function
      2. Video presentations/backgrounds
         1. Video styles
      3. Distracting to have unrelated tabs at top
      4. Text wrapping in window view
      5. Images
      6. Change organization and order of modules
      7. Orient the reader
      8. Course progression
         1. Login system to record course progression
         2. Option for sequential progression
      9. Accessibility
      10. Inclusivity
      11. Additional features
          1. Downloadable summaries
          2. Contact details
          3. Completion page
             1. Certificate of completion
          4. Real-time learning opportunities
      12. UQ branded content only
      13. Advanced course options
5. Confidence reviewing/discussing genomics applications
   1. Improved
   2. Did not improve
6. Recommendation to others and comments
   1. Willingness to recommend to other members
      1. Would recommend
         1. Non-HREC uses
      2. Would not recommend
   2. Overall recommendations/comments
      1. Positive
7. Illustrative quotes
